# COVID-19-Induced Myopericarditis Leading to Cardiac Tamponade: An Unusual Case Presentation

**DOI:** 10.7759/cureus.27158

**Published:** 2022-07-22

**Authors:** Niel Shah, Mohamed Saleh, Abhilasha Jyala, Vibha Hayagreev, Muhammad Saad

**Affiliations:** 1 Internal Medicine, Einstein Medical Center Philadelphia, Philadelphia, USA; 2 Internal Medicine, The Ohio State University Wexner Medical Center, Columbus, USA; 3 Internal Medicine, BronxCare Health System, Bronx, USA; 4 Cardiology, BronxCare Health System/Icahn School of Medicine at Mount Sinai, Bronx, USA

**Keywords:** pericardial effusion, viral pericarditis, coronavirus pandemic, myopericarditis, cardiac tamponade, covid 19

## Abstract

Coronavirus disease 2019 (COVID-19) can manifest differently in different patients, ranging from asymptomatic carriers to acute respiratory distress syndrome (ARDS). Cardiac involvement may occur with COVID-19 even without respiratory tract signs and symptoms of infection. Cardiac manifestations like heart failure (HF), myopericarditis, and cardiac arrhythmias are commonly reported. Cardiac injury with troponin leak is associated with increased mortality in COVID-19, and its clinical and radiographic features are difficult to distinguish from those of HF. COVID-19 is also known to cause pericardial inflammation, likely via direct cytotoxic effects or immune-mediated mechanisms. However, the definite mechanism is still unclear. We present here a case of myopericarditis complicated by pericardial effusion and cardiac tamponade in a COVID-19 infected patient with minimal pulmonary involvement.

## Introduction

The coronavirus disease 2019 (COVID-19) pandemic began in December 2019 and continues spreading rapidly worldwide [1]. It is caused by the novel SARS-CoV-2. The disease presentation can vary anywhere from being an asymptomatic carrier to acute respiratory distress syndrome (ARDS). SARS-CoV-2 enters the human host by binding to the ACE2 receptor, which is expressed in the lung and heart. Cardiac involvement may occur with COVID-19 even without symptoms and signs of respiratory involvement. Cardiac manifestations like heart failure (HF), myopericarditis, and cardiac arrhythmias are commonly reported [2-8], but there is limited literature available on cardiac tamponade [5]. Pericardial effusion is associated with severe COVID-19, and it has been reported that up to 7% of COVID-19-related deaths were attributable to myocarditis and its complications [4]. However, this was based on assumption and not on a confirmatory diagnosis of myocarditis and thus may be overestimated. Cardiac injury with troponin leak is associated with increased mortality in COVID-19, and its clinical and radiographic features are difficult to distinguish from those of HF [2]. In one meta-analysis, approximately 5% of patients with COVID-19 who underwent CT chest had a detectable pericardial effusion. Patients with previous cardiac disease and/or a structurally abnormal heart may be at increased risk of developing COVID-19 myopericarditis and cardiac tamponade. It has been proposed that COVID-19 causes pericardial inflammation via direct cytotoxic effects or immune-mediated mechanisms, but the definite mechanism is unclear. We describe a case of myopericarditis complicated by pericardial effusion and cardiac tamponade in a COVID-19-infected patient with minimal pulmonary involvement.

## Case presentation

A 64-year-old Hispanic woman presented to the ED with complaints of chest pain that started two weeks ago. She described it as retrosternal, intermittent, fluctuating between 5 and 9 out of 10 in intensity, radiating to the back, associated with sweating, and exacerbated with movements. She complained of excessive fatigue, malaise, decreased appetite for the same duration, and dyspnea on exertion. Her exercise tolerance was 5-6 blocks and was limited due to dyspnea on exertion. The patient also complained of left lower abdominal pain for the past week, which was crampy and was associated with non-bloody diarrhea and episodes of nausea. She denied cough, fever, chills, recent travel, or sick contact exposure. The review of the system was negative otherwise. Her medical history was significant for diabetes mellitus, hypertension, and hyperlipidemia. She had a surgical history of open appendectomy, cesarian section, and cholecystectomy. She denied a history of alcohol abuse, smoking, or illicit drug use. Physical exam revealed distant heart sounds, S3 gallop, elevated jugular venous pulse (JVP), and positive hepatojugular reflex. Otherwise, the physical exam was unremarkable.

**Figure 1:**
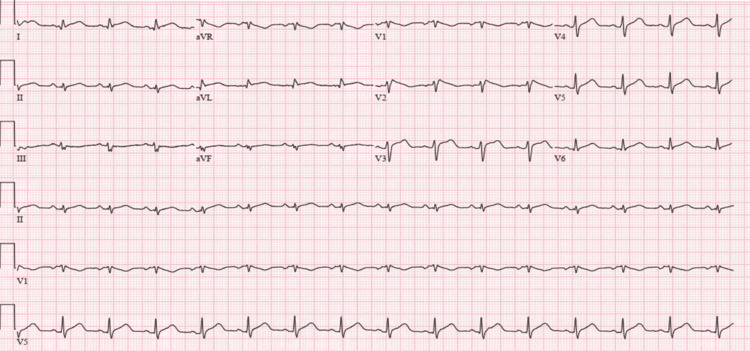
ECG showing normal sinus rhythm and low voltage QRS complexes.

**Figure 2:**
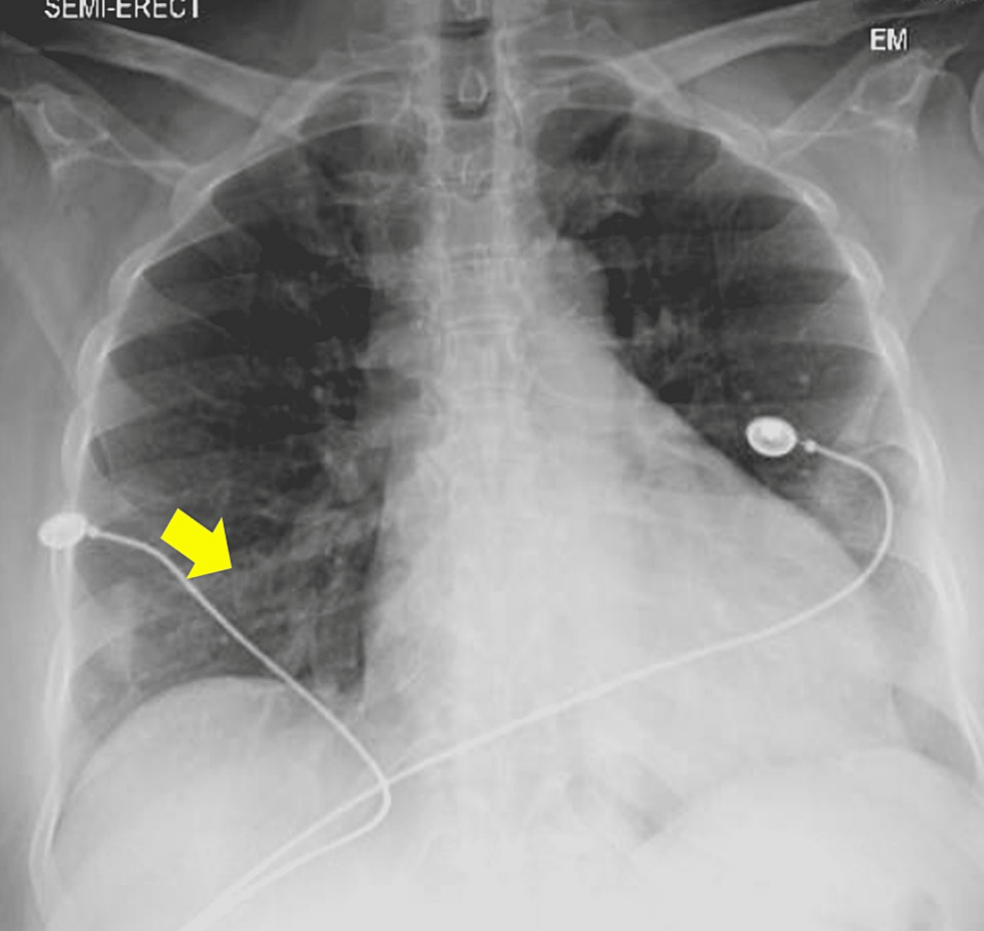
Chest X-ray showing nodular densities in the right lower lung field (yellow arrow).

**Figure 3:**
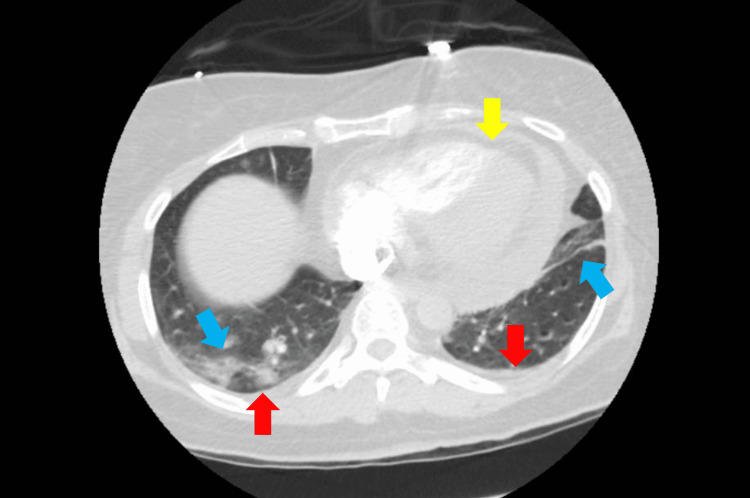
CT scan of the chest with contrast showed moderate pericardial effusion (yellow arrow), bilateral patchy infiltrates (blue arrow), and trace bilateral pleural effusion (red arrow).

In the ED, the patient was tachycardic and hypotensive. She was given a trial of IV fluids with improvement in blood pressure. Initial ECG showed atrial fibrillation with a rapid ventricular response, low voltage QRS complexes (Figure 1), and diffuse ST-segment elevation. Laboratory tests were significant for leukocytosis, elevated C-reactive protein (CRP, 40 mg/L), elevated ferritin, transaminitis, indirect hyperbilirubinemia, acute kidney injury, elevated troponin, elevated pro B type natriuretic peptide (proBNP), and lactic acidosis. The patient was found to be COVID-19 positive. Also, the patient was found to be anti-SARS-CoV-2 antibodies positive, which represents the late presentation to the hospital. Chest X-ray (CXR) showed nodular densities in the right lower lobe (Figure 2). Bedside ultrasound showed mild pericardial effusion, enlarged right ventricle with diastolic collapse, and dilated inferior vena cava (IVC) suggestive of cardiac tamponade. The patient was admitted to the cardiac ICU for further management of cardiac tamponade in the setting of COVID-19-related myopericarditis. The patient was started on IV fluids and colchicine. Pneumonia and septic workup were sent, and the patient was started on broad-spectrum antibiotics. CT scan of the chest with contrast was done, and pulmonary embolism (PE) was ruled out. However, it showed moderate pericardial effusion, bilateral patchy infiltrates, and trace bilateral pleural effusion (Figure 3).

**Figure 4:**
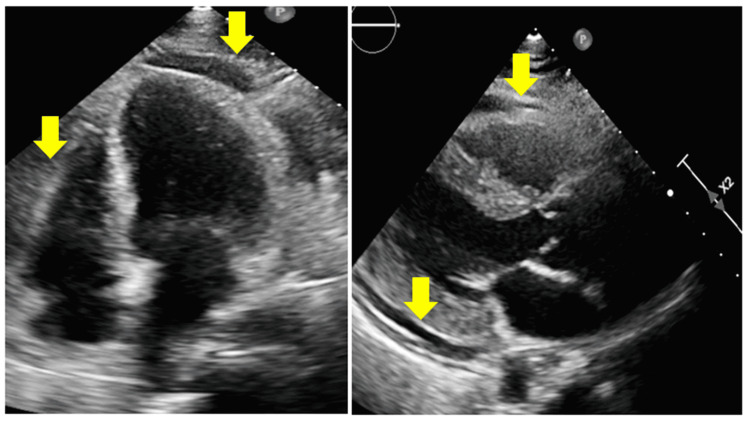
Initial echocardiographic findings showing RV diastolic collapse and moderate-to-large pericardial effusion suggestive of cardiac tamponade (yellow arrows).

**Figure 5:**
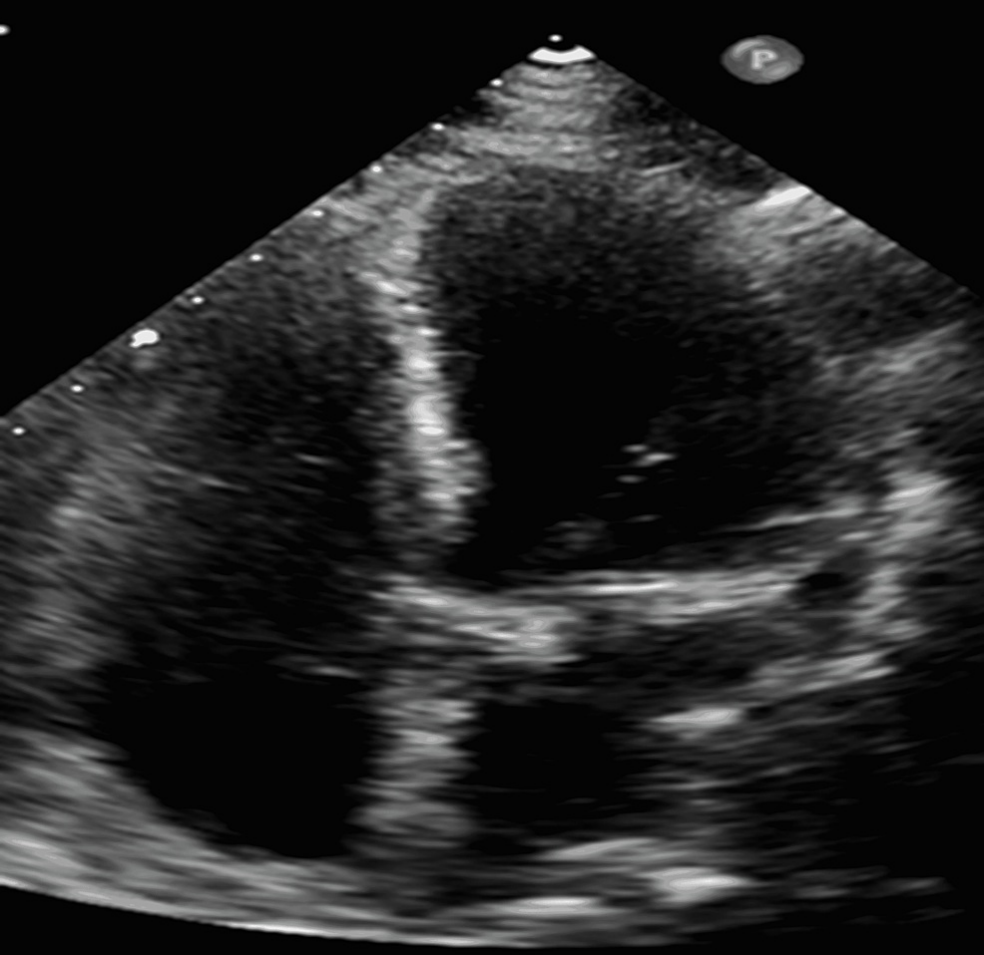
Echocardiogram findings after pericardiocentesis showing trivial pericardial effusion without signs of tamponade and improvement in LVEF. LVEF: Left ventricular ejection fraction.

In the cardiac ICU, the patient was started on corticosteroids and IV immunoglobulin (IVIG) for the possible multisystem inflammatory syndrome as per infectious diseases (ID) specialist's recommendations. Transthoracic echocardiogram (Figure 4) showed left ventricular ejection fraction (LVEF) of 36% and large fibrinous fluid localized around right ventricle 2.38 cm with the diastolic collapse of RV free wall, moderate fluid around right atrium (1.2 cm) and moderate fluid collection posterior of the left ventricle with echocardiographic signs of cardiac tamponade. Also, it showed dilated IVC and hepatic veins compliant with cardiac tamponade. The patient underwent pericardiocentesis, and 300 cc of serous pericardial fluid was drained. Pericardial fluid analysis showed yellow, cloudy fluid with neutrophil predominance. Other tests from pericardial fluid such as bacterial cultures, adenosine deaminase (ADA), and mycobacterial cultures were negative. Autoimmune workup was also unremarkable. COVID-19 polymerase chain reaction (PCR) test from pericardiac fluid was negative. Considering all the negative workup, the etiology for pericarditis and pericardial effusion was suspected to be COVID-19 infection. Post pericardiocentesis echocardiogram (Figure 5) showed improvement in LVEF at 48% and trivial pericardial effusion without signs of tamponade. ID closely followed the patient and recommended completing five days of antibiotics and 10 days of steroids. Atrial fibrillation was converted to normal sinus rhythm spontaneously during the hospital course without any further episodes of atrial fibrillation. Thus the patient was not a candidate for anticoagulation as per cardiology recommendations. During the hospital course, the patient showed clinical improvement while taking steroids and colchicine. An echocardiogram prior to discharge showed trivial pericardial effusion without signs of cardiac tamponade. The patient was discharged on colchicine for three months and steroids for 10 days. Also, the patient was started on goal-directed medical treatment for HF with reduced EF. The patient followed up in the cardiology clinic, and she was doing well post-discharge.

## Discussion

COVID-19 has various cardiovascular complications. Cardiac tissue pathology with troponin leak is associated with higher mortality in COVID-19 disease. Data regarding cardiovascular involvement due to SARS-CoV-2 infection are less described [2,6,9]. Although the spectrum of clinical presentation is directly connected to the inflammatory process of the respiratory tract, our case highlights evidence of cardiac involvement as a potential late phenomenon of the viral infection. This process can be subclinical, reported by an autopsy study, or may present with overt manifestations or even minimal respiratory involvement, as in our case [10].

Acute pericarditis is the commonest pathology of the pericardium and is responsible for about 0.2% of chest pain-related hospitalizations. In general, the clinical data on the etiology of myopericarditis suggest that viral infections are possibly the most common causes in developed countries. Commonly involved viruses include Coxsackieviruses (especially Coxsackie B), Herpes viruses, Influenza virus, and Parvovirus B19 [11]. It is hypothesized that COVID-19, similar to other viruses, causes pericardial inflammation via direct cytotoxic effects or immune-mediated mechanisms; however, the exact mechanism is unclear [12]. However, to our knowledge, only a few cases of myopericarditis and cardiac tamponade are linked with the SARS-CoV-2 infection [5,6,13]. The diagnosis of myopericarditis is usually based on a characteristic history of pleuritic-nature chest pain, diagnostic criteria for pericarditis (i.e., pericardial rubs, widespread ST-segment elevation, and pericardial effusion), and troponin elevation [14]. Our patient presented to the ED after two weeks of symptoms. In addition, COVID-19 antibodies were positive at the time of admission, suggesting a late admission time in the course of the disease. Interestingly, the patient in this case presented with chest pain and abdominal symptoms as the leading cause of seeking medical advice. The patient saturated 97-100% on room air during most of her admission period with occasional use of a nasal cannula, limited to 1-2 liters at maximum. 

Presently, non-steroidal anti-inflammatory drugs (NSAIDs) are the mainstay of anti-inflammatory therapy for acute pericarditis and myopericarditis, together with the use of colchicine [15]. In case of failure, intolerance, or contraindication to NSAID, corticosteroids at low-to-medium doses (e.g., prednisone 0.2-0.5 mg/kg/day) are indicated as the second-line option in combination with colchicine [12,15]. In patients with refractory recurrent pericarditis, despite NSAIDs, corticosteroids, and colchicine, third-line therapies are recommended, including IVIG, azathioprine, or anti-IL (e.g., anakinra) [15-17]. Pericardiocentesis remains an option for patients with severe symptoms of cardiac tamponade. Our patient received colchicine, IVIG, and corticosteroids and eventually underwent pericardiocentesis for cardiac tamponade.

Currently, our understanding of the transmission, pathology, spectrum of clinical presentation, and therapeutic options for COVID-19 is expanding. Cardiac involvement is established to be a significant component of the pathological picture of COVID-19 disease. This case highlights the value of understanding COVID-19 infection with atypical clinical presentations such as myopericarditis, pericardial effusion, cardiac tamponade, and if there is any need for early hospitalization. This case report is helpful in treating patients with this unique clinical presentation.

## Conclusions

Our case highlights evidence of cardiac involvement as a potential late phenomenon of the viral infection with minimal lung involvement. COVID-19 causes pericardial inflammation via direct cytotoxic effects or immune-mediated mechanisms, but the definite mechanism is unclear. Only a few cases of myopericarditis and cardiac tamponade are known that are linked with the SARS-CoV-2 infection to date, including our case. The diagnosis of myopericarditis can usually be made based on the combination of history taking (pleuritic chest pain), physical exam (pericardial rubs), findings of diagnostic modalities (widespread ST-segment elevation and echocardiographic findings), and elevated troponin levels. NSAIDs, colchicine, or steroids remain the mainstays of the treatment for myopericarditis, while pericardiocentesis is the gold standard treatment for cardiac tamponade. Our case highlights the clinical picture and management of the atypical clinical presentations associated with COVID-19, such as myopericarditis, pericardial effusion, and cardiac tamponade.
